# 5-(4-Hy­droxy­phen­yl)imidazolidine-2,4-dione

**DOI:** 10.1107/S1600536814010034

**Published:** 2014-05-10

**Authors:** Jin-hui Xue, Yong-qi Qin, Zi-feng Zhang, Yuan-biao Qiao

**Affiliations:** a Department of Chemistry and Chemical Engineering, Lvliang University, Lvliang, Shanxi 033001, People’s Republic of China; b Laboratory of Medicinal Chemistry, Lvliang University, Lvliang, Shanxi 033001, People’s Republic of China

## Abstract

The title compound, C_9_H_8_N_2_O_3_, was prepared by reaction of phenol, glyoxylic acid and urea in water. The imidazolidine ring adopts an almost planar conformation (r.m.s. deviation = 0.012 Å) and is twisted by 89.3 (1)° relative to the benzene ring. In the crystal, mol­ecules are linked by N—H⋯O and O—H⋯O hydrogen bonds into a three-dimensional framework.

## Related literature   

For general background to the synthesis and applications of hydantoin derivatives, see: Liu & Zhao (2001 ▶); Dhar *et al.* (2002 ▶); Goodnow & Kang (2003 ▶). For related compounds, see: Ji *et al.* (2002 ▶).
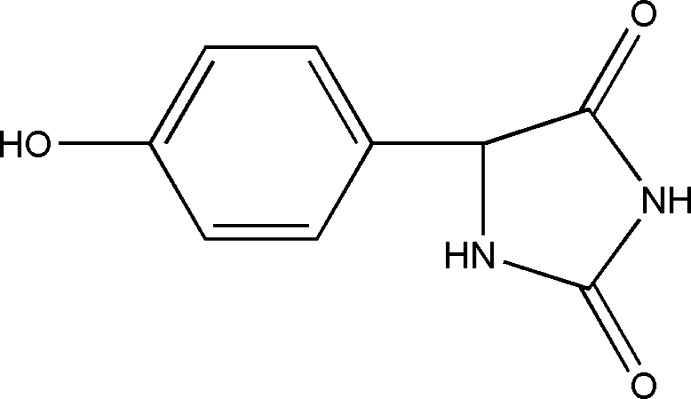


## Experimental   

### 

#### Crystal data  

C_9_H_8_N_2_O_3_
*M* *_r_* = 192.17
Monoclinic, 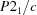

*a* = 10.3694 (11) Å
*b* = 6.9914 (8) Å
*c* = 12.3857 (13) Å
β = 105.619 (2)°
*V* = 864.76 (16) Å^3^
*Z* = 4
Mo *K*α radiation
μ = 0.11 mm^−1^
*T* = 296 K
0.30 × 0.20 × 0.20 mm

#### Data collection  

Bruker SMART CCD area-detector diffractometer
4721 measured reflections
1558 independent reflections
1100 reflections with *I* > 2σ(*I*)
*R*_int_ = 0.035

#### Refinement  

*R*[*F* ^2^ > 2σ(*F* ^2^)] = 0.035
*wR*(*F* ^2^) = 0.091
*S* = 1.02
1558 reflections
137 parameters
H atoms treated by a mixture of independent and constrained refinement
Δρ_max_ = 0.15 e Å^−3^
Δρ_min_ = −0.14 e Å^−3^

### 

Data collection: *SMART* (Bruker, 1997 ▶); cell refinement: *SAINT* (Bruker, 1997 ▶); data reduction: *SAINT*; program(s) used to solve structure: *SHELXS97* (Sheldrick, 2008 ▶); program(s) used to refine structure: *SHELXL2013* (Sheldrick, 2008 ▶); molecular graphics: *SHELXTL* (Sheldrick, 2008 ▶); software used to prepare material for publication: *SHELXTL*.

## Supplementary Material

Crystal structure: contains datablock(s) I. DOI: 10.1107/S1600536814010034/kq2013sup1.cif

Structure factors: contains datablock(s) I. DOI: 10.1107/S1600536814010034/kq2013Isup2.hkl

Supporting information file. DOI: 10.1107/S1600536814010034/kq2013Isup3.cml

CCDC reference: 1000728

Additional supporting information:  crystallographic information; 3D view; checkCIF report

## Figures and Tables

**Table 1:** Hydrogen-bond geometry (Å, °)

*D*—H⋯*A*	*D*—H	H⋯*A*	*D*⋯*A*	*D*—H⋯*A*
N2—H2⋯O4^i^	0.883 (18)	1.952 (19)	2.8180 (19)	166.7 (17)
N1—H1⋯O4^ii^	0.85 (2)	2.535 (19)	3.204 (2)	136.5 (16)
N1—H1⋯O6^iii^	0.85 (2)	2.36 (2)	3.067 (2)	141.1 (17)
O6—H6⋯O5^iv^	0.95 (2)	1.78 (2)	2.7223 (18)	169.0 (18)

Symmetry codes: (i) 
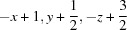
; (ii) 
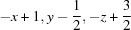
; (iii) 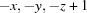; (iv) 
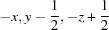
.

**Fig. 1.**
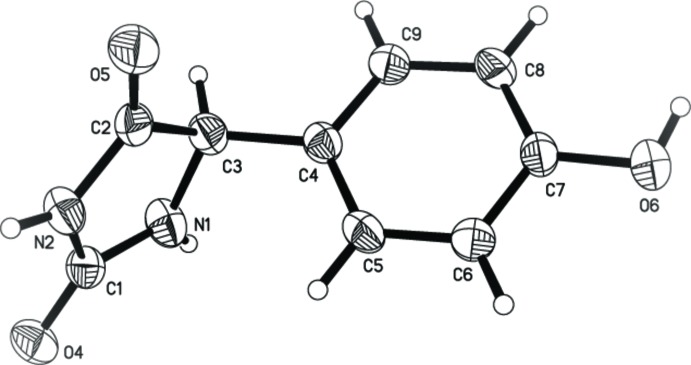
Molecular structure of I. Displacement ellipsoids are presented at the 40% probability level. H atoms are depicted as small spheres of arbitrary radius.

**Fig. 2.**
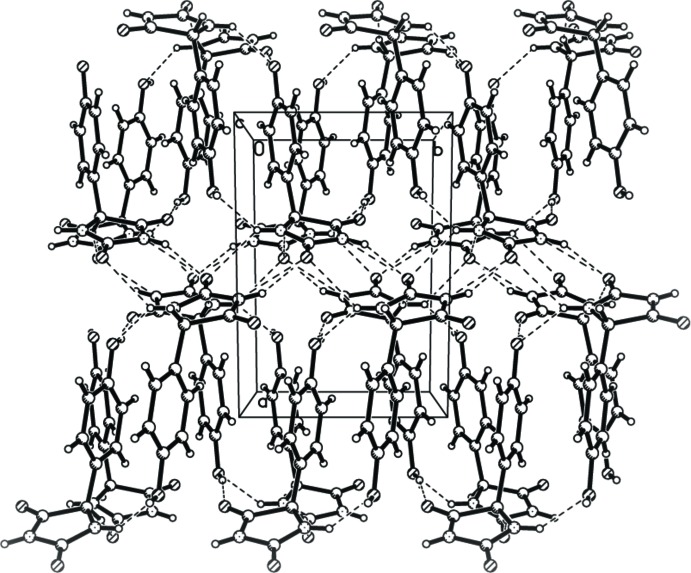
A portion of the crystal structure of I viewed along [001]. The intermolecular hydrogen bonding interactions are depicted by dashed lines.

## supplementary crystallographic information

### 1. Comment

Hydantoin derivatives can be used as intermediates in pharmaceutical products, pesticides and photosensitive material. It is very important to the development of hydantoins compounds. Pharmacological functions of hydantoin derivatives are mainly shown in antibacterial (Liu & Zhao, 2001), diminishing inflammation (Dhar *et al.*, 2002), relieving cough and asthma, lowering blood sugar (Goodnow & Kang, 2003), and inhibiting agent of uremic toxin. Different substituted hydantoin and its derivatives show good application future, such as the treatment of diabetes, kidney disease, autoimmune disease and blood disease. The spectrum of hydantoin derivatives is broad as bacterial disinfectant. They are widely used in aquaculture, pest and disease control, disinfection treatment of health equipment, mildew prevention and control of crops, preservation of vegetable & Fruit, and mildew anti-corrosion of industrial products and living goods.

In the molecule of the title compound, C_9_H_8_N_2_O_3_, I (Fig. 1) bond lengths and angles are generally normal (Ji *et al.*, 2002). The imidazolidine ring adopts a planar conformation (r.m.s. deviation is 0.012 Å) and is twisted by 89.3 (1)° relative to the benzene plane.

In the crystal, molecules are bound by intermolecular N—H···O and O—H···O hydrogen bonds (Table 1) into three-dimensional framework (Fig. 2).

### 2. Experimental

The title compound was prepared by reaction of phenol (0.05 mol), glyoxylic acid (0.06 mol) and urea (0.06 mol) in hydrochloric acid (37%, 80 ml) at 370 K for 6 h, cooling, filtering, affording the tile compound by recrystallization in water. Single crystals of the title compound suitable for X-ray measurements was obtained by recrystallization from ethanol at room temperature.

### 3. Refinement

The hydroxyl and amino hydrogen atoms were objectively localized in the difference-Fourier map and refined isotropically with fixed displacement parameters. The other hydrogen atoms were placed in the calculated positions with C—H distances = 0.93–0.98 Å and refined in the riding model with fixed isotropic displacement parameters: *U*_iso_(H) = 1.2*U*_eq_(C).

### Crystal data

**Table:**

C_9_H_8_N_2_O_3_	*F*(000) = 400
*M**_r_* = 192.17	*D*_x_ = 1.476 Mg m^−^^3^
Monoclinic, *P*2_1_/*c*	Mo *K*α radiation, λ = 0.71073 Å
*a* = 10.3694 (11) Å	Cell parameters from 933 reflections
*b* = 6.9914 (8) Å	θ = 2.0–25.0°
*c* = 12.3857 (13) Å	µ = 0.11 mm^−^^1^
β = 105.619 (2)°	*T* = 296 K
*V* = 864.76 (16) Å^3^	Rectangle, colourless
*Z* = 4	0.30 × 0.20 × 0.20 mm

### Data collection

**Table:**

Bruker SMART CCD area-detector diffractometer	1100 reflections with *I* > 2σ(*I*)
Radiation source: fine-focus sealed tube	*R*_int_ = 0.035
Graphite monochromator	θ_max_ = 25.2°, θ_min_ = 2.0°
phi and ω scans	*h* = −12→12
4721 measured reflections	*k* = −8→8
1558 independent reflections	*l* = −7→14

### Refinement

**Table:**

Refinement on *F*^2^	Secondary atom site location: difference Fourier map
Least-squares matrix: full	Hydrogen site location: difference Fourier map
*R*[*F*^2^ > 2σ(*F*^2^)] = 0.035	H atoms treated by a mixture of independent and constrained refinement
*wR*(*F*^2^) = 0.091	*w* = 1/[σ^2^(*F*_o_^2^) + (0.0426*P*)^2^ + 0.0206*P*] where *P* = (*F*_o_^2^ + 2*F*_c_^2^)/3
*S* = 1.02	(Δ/σ)_max_ < 0.001
1558 reflections	Δρ_max_ = 0.15 e Å^−^^3^
137 parameters	Δρ_min_ = −0.14 e Å^−^^3^
0 restraints	Extinction correction: *SHELXL2013* (Sheldrick, 2008), Fc^*^=kFc[1+0.001xFc^2^λ^3^/sin(2θ)]^-1/4^
Primary atom site location: structure-invariant direct methods	Extinction coefficient: 0.024 (3)

### Special details

**Table:**

Geometry. All e.s.d.'s (except the e.s.d. in the dihedral angle between two l.s. planes) are estimated using the full covariance matrix. The cell e.s.d.'s are taken into account individually in the estimation of e.s.d.'s in distances, angles and torsion angles; correlations between e.s.d.'s in cell parameters are only used when they are defined by crystal symmetry. An approximate (isotropic) treatment of cell e.s.d.'s is used for estimating e.s.d.'s involving l.s. planes.

### Fractional atomic coordinates and isotropic or equivalent isotropic displacement parameters (Å^2^)

**Table:**

	*x*	*y*	*z*	*U*_iso_*/*U*_eq_	
N1	0.36582 (16)	0.1852 (2)	0.60788 (13)	0.0438 (4)	
H1	0.3651 (18)	0.070 (3)	0.6298 (15)	0.053*	
N2	0.39496 (14)	0.4952 (2)	0.61795 (12)	0.0394 (4)	
H2	0.4272 (18)	0.605 (3)	0.6494 (15)	0.047*	
O4	0.46179 (12)	0.32043 (18)	0.78108 (10)	0.0464 (4)	
O5	0.32237 (12)	0.58433 (19)	0.43383 (10)	0.0508 (4)	
O6	−0.23743 (12)	0.1408 (2)	0.29172 (11)	0.0512 (4)	
H6	−0.2595 (19)	0.108 (3)	0.2145 (17)	0.061*	
C1	0.41256 (16)	0.3268 (3)	0.67946 (15)	0.0360 (4)	
C2	0.34366 (16)	0.4631 (3)	0.50733 (15)	0.0362 (4)	
C3	0.31532 (17)	0.2501 (2)	0.49270 (14)	0.0385 (5)	
H3	0.3691	0.1943	0.4466	0.046*	
C4	0.16868 (17)	0.2098 (2)	0.43983 (14)	0.0353 (4)	
C5	0.07338 (17)	0.2567 (3)	0.49525 (15)	0.0410 (5)	
H5	0.1009	0.3060	0.5676	0.049*	
C6	−0.06081 (18)	0.2319 (3)	0.44558 (15)	0.0417 (5)	
H6A	−0.1233	0.2633	0.4842	0.050*	
C7	−0.10245 (17)	0.1601 (2)	0.33802 (14)	0.0366 (4)	
C8	−0.00956 (17)	0.1091 (3)	0.28186 (14)	0.0413 (5)	
H8	−0.0374	0.0583	0.2099	0.050*	
C9	0.12569 (18)	0.1341 (3)	0.33336 (14)	0.0414 (5)	
H9	0.1883	0.0992	0.2955	0.050*	

### Atomic displacement parameters (Å^2^)

**Table:**

	*U*^11^	*U*^22^	*U*^33^	*U*^12^	*U*^13^	*U*^23^
N1	0.0489 (10)	0.0338 (9)	0.0412 (10)	−0.0001 (8)	−0.0011 (7)	0.0047 (7)
N2	0.0423 (9)	0.0360 (9)	0.0336 (9)	−0.0046 (7)	−0.0009 (7)	0.0005 (7)
O4	0.0472 (8)	0.0544 (9)	0.0312 (8)	0.0075 (6)	−0.0002 (6)	0.0051 (6)
O5	0.0534 (9)	0.0547 (9)	0.0394 (8)	−0.0061 (7)	0.0038 (6)	0.0111 (7)
O6	0.0360 (8)	0.0701 (10)	0.0427 (8)	−0.0081 (6)	0.0023 (6)	−0.0054 (7)
C1	0.0277 (9)	0.0427 (11)	0.0348 (11)	0.0036 (8)	0.0035 (8)	0.0016 (8)
C2	0.0281 (9)	0.0437 (11)	0.0340 (11)	−0.0025 (8)	0.0036 (8)	0.0027 (9)
C3	0.0357 (10)	0.0431 (11)	0.0342 (10)	0.0011 (8)	0.0054 (8)	−0.0028 (8)
C4	0.0374 (10)	0.0326 (10)	0.0338 (10)	−0.0025 (8)	0.0063 (8)	−0.0021 (8)
C5	0.0433 (12)	0.0468 (11)	0.0310 (10)	−0.0035 (9)	0.0068 (8)	−0.0100 (8)
C6	0.0394 (11)	0.0483 (12)	0.0385 (11)	−0.0017 (9)	0.0126 (8)	−0.0076 (9)
C7	0.0330 (10)	0.0377 (11)	0.0359 (10)	−0.0036 (8)	0.0040 (8)	0.0007 (8)
C8	0.0460 (11)	0.0463 (11)	0.0297 (10)	−0.0078 (9)	0.0071 (8)	−0.0090 (8)
C9	0.0399 (11)	0.0489 (12)	0.0368 (11)	−0.0028 (9)	0.0124 (8)	−0.0078 (9)

### Geometric parameters (Å, º)

**Table:**

N1—C1	1.330 (2)	C3—H3	0.9800
N1—C3	1.454 (2)	C4—C9	1.379 (2)
N1—H1	0.85 (2)	C4—C5	1.386 (3)
N2—C2	1.348 (2)	C5—C6	1.373 (2)
N2—C1	1.387 (2)	C5—H5	0.9300
N2—H2	0.883 (18)	C6—C7	1.380 (2)
O4—C1	1.2251 (18)	C6—H6A	0.9300
O5—C2	1.220 (2)	C7—C8	1.378 (3)
O6—C7	1.3690 (19)	C8—C9	1.387 (2)
O6—H6	0.95 (2)	C8—H8	0.9300
C2—C3	1.519 (2)	C9—H9	0.9300
C3—C4	1.511 (2)		
			
C1—N1—C3	113.08 (15)	C9—C4—C5	118.33 (16)
C1—N1—H1	121.6 (13)	C9—C4—C3	120.95 (17)
C3—N1—H1	125.3 (13)	C5—C4—C3	120.65 (16)
C2—N2—C1	111.98 (15)	C6—C5—C4	121.34 (17)
C2—N2—H2	126.3 (12)	C6—C5—H5	119.3
C1—N2—H2	121.0 (12)	C4—C5—H5	119.3
C7—O6—H6	112.8 (12)	C5—C6—C7	119.65 (17)
O4—C1—N1	129.33 (17)	C5—C6—H6A	120.2
O4—C1—N2	123.50 (17)	C7—C6—H6A	120.2
N1—C1—N2	107.17 (15)	O6—C7—C8	122.53 (16)
O5—C2—N2	125.82 (17)	O6—C7—C6	117.33 (16)
O5—C2—C3	127.01 (16)	C8—C7—C6	120.13 (15)
N2—C2—C3	107.16 (15)	C7—C8—C9	119.54 (16)
N1—C3—C4	114.94 (15)	C7—C8—H8	120.2
N1—C3—C2	100.48 (13)	C9—C8—H8	120.2
C4—C3—C2	111.97 (14)	C4—C9—C8	120.98 (17)
N1—C3—H3	109.7	C4—C9—H9	119.5
C4—C3—H3	109.7	C8—C9—H9	119.5
C2—C3—H3	109.7		
			
C3—N1—C1—O4	−179.77 (17)	C2—C3—C4—C9	112.19 (19)
C3—N1—C1—N2	0.8 (2)	N1—C3—C4—C5	49.1 (2)
C2—N2—C1—O4	177.58 (16)	C2—C3—C4—C5	−64.7 (2)
C2—N2—C1—N1	−3.0 (2)	C9—C4—C5—C6	−1.0 (3)
C1—N2—C2—O5	−177.36 (17)	C3—C4—C5—C6	175.96 (17)
C1—N2—C2—C3	3.78 (19)	C4—C5—C6—C7	−0.5 (3)
C1—N1—C3—C4	−119.10 (17)	C5—C6—C7—O6	−179.04 (16)
C1—N1—C3—C2	1.27 (19)	C5—C6—C7—C8	1.6 (3)
O5—C2—C3—N1	178.20 (17)	O6—C7—C8—C9	179.43 (16)
N2—C2—C3—N1	−2.96 (17)	C6—C7—C8—C9	−1.3 (3)
O5—C2—C3—C4	−59.3 (2)	C5—C4—C9—C8	1.4 (3)
N2—C2—C3—C4	119.51 (16)	C3—C4—C9—C8	−175.60 (17)
N1—C3—C4—C9	−133.99 (17)	C7—C8—C9—C4	−0.2 (3)

### Hydrogen-bond geometry (Å, º)

**Table:**

*D*—H···*A*	*D*—H	H···*A*	*D*···*A*	*D*—H···*A*
N2—H2···O4^i^	0.883 (18)	1.952 (19)	2.8180 (19)	166.7 (17)
N1—H1···O4^ii^	0.85 (2)	2.535 (19)	3.204 (2)	136.5 (16)
N1—H1···O6^iii^	0.85 (2)	2.36 (2)	3.067 (2)	141.1 (17)
O6—H6···O5^iv^	0.95 (2)	1.78 (2)	2.7223 (18)	169.0 (18)

Symmetry codes: (i) −*x*+1, *y*+1/2, −*z*+3/2; (ii) −*x*+1, *y*−1/2, −*z*+3/2; (iii) −*x*, −*y*, −*z*+1; (iv) −*x*, *y*−1/2, −*z*+1/2.

## References

[bb1] Bruker (1997). *SMART* and *SAINT* Bruker AXS Inc., Madison, Wisconsin, USA.

[bb2] Dhar, T. G., Iwanowicz, E., Launay, M., Maillet, M., Nicolai, E. & Potin, D. (2002). *Hydantoin compounds useful as anti-inflammatory agents* US Patent US2002/0143035A1[P].

[bb3] Goodnow, J. R. & Kang, L. (2003). *Hydantoin-containing glucokinase activators* US Patent US2003/0225286A1[P].

[bb4] Ji, B. M., Du, C. X., Zhu, Y. & Wang, Y. (2002). *Chin. J. Struct. Chem.* **21**, 252–255.

[bb5] Liu, J. & Zhao, Y. (2001). *Chin. J. Disinfect.* **18**, 218–222.

[bb6] Sheldrick, G. M. (2008). *Acta Cryst.* A**64**, 112–122. 10.1107/S0108767307043930 18156677

